# Knowledge, Attitude, and Practices to Foodborne Zoonotic Diseases and Their Associated Factors in and Around Debre Tabor City, Northwest Ethiopia

**DOI:** 10.1155/vmi/8360480

**Published:** 2025-03-25

**Authors:** Balemual Abebaw, Sisay Assefie

**Affiliations:** ^1^Department of Veterinary Science, College of Agriculture and Environmental Science, Debre Tabor University, Debra Tabor, Ethiopia; ^2^Department of Public Health, College of Medicine and Health Sciences, University of Gondar, Gondar, Ethiopia

**Keywords:** awareness, Debre Tabor city, foodborne infection, knowledge, practice

## Abstract

**Background:** Foodborne zoonotic diseases are a widespread public health problem globally. Infections are naturally transmitted between animals and humans through food.

**Materials and Methods:** A cross-sectional study was conducted from January to June 2024 to evaluate the awareness of foodborne zoonotic diseases and their associated factors in and around Debre Tabor city, Northwest Ethiopia. In this study, 771 participants were involved in which, a single individual over the age of 18 was chosen from each family using a simple random sampling method for data collection. Data were processed using Epi Info Version 7, and the analysis was conducted with SPSS Version 20 software. Bivariable and multivariable logistic regression analyses were utilized to examine the influence of different factors on the dependent variable's level.

**Results:** A total of 771 participants took part in the study, resulting in an overall response rate of 95.7%. The number of participants having a good knowledge and practice towards meat-borne zoonoses were 378 (49%) and 412 (58.6%), respectively. The knowledge of the respondents on tuberculosis, anthrax, taeniasis, and cysticercosis was 192 (24.9%), 335 (43.5%), 313 (40.6%), and 266 (34.5%), respectively. Educational status and access to information sources of respondents were significantly associated with knowledge as *p* value < 0.05 at a 95% confidence interval.

**Conclusion:** The level of knowledge and practice was poor about foodborne zoonotic diseases in this study. This is because of the habit of backyard slaughtering and consumption of raw meat.

## 1. Introduction

Foodborne diseases have major public health effects and are a widespread problem globally. Developing nations bear the brunt of issues caused by foodborne illnesses [1].

In industrialized nations, as many as 10% of people may experience foodborne zoonotic illnesses each year. Foodborne zoonotic diseases are naturally transmissible indirectly between animals and humans through food [2]. The World Health Organization (WHO) and the US Centers for Disease Control and Prevention have reported that numerous individuals have been infected due to food contamination. The majority of pathogens involved in foodborne zoonotic diseases originate from animals and are found in healthy livestock [3].

Animal-derived foods are the most widely eaten sources of nutrition among communities globally. However, if they are not managed and prepared correctly, they can result in numerous foodborne illnesses [4]. Foodborne zoonotic diseases are caused by parasites, bacteria, and viruses. International food trade is increasing the chance of the spread of disease around the world more easily [5].

The World Organization for Animal Health (OIE), Food and Agriculture Organization (FAO), and WHO collaboration related to foodborne zoonotic diseases plays a vital role in public health importance [6].

Foodborne zoonotic diseases are among the top causes of illness and mortality in humans across the globe [7, 8]. The 1982 outbreak of hemorrhagic colitis was associated with the consumption of undercooked ground beef [9]. Meat has been used as a vehicle for a significant proportion of human foodborne diseases [10].

The challenge of foodborne zoonotic diseases is more serious among rural communities because of their low level of awareness and poor hygiene environment [11]. Various management systems and practices within the community of smallholder livestock-keeping in rural and periurban regions can affect the likelihood of zoonotic diseases [12]. Tapeworms, anthrax, and bovine tuberculosis (BTB) are among the most common foodborne zoonoses that infect humans and animals [13].

*Taenia saginata* infections are common in Ethiopia because of the prevalent practice of consuming raw meat and the tendency to defecate in open grazing areas. Infections in Ethiopia remain unreported. This is a result of self-medication with either contemporary or traditional herbal remedies [14]. Bovine cysticercosis is an infection in cattle caused by *Cysticercus bovis*, the larval form of *T. saginata*. Consuming raw or undercooked meat leads to the occurrence of taeniasis in humans. It significantly contributes to economic loss primarily because of the condemnation and reduction in quality of infected carcasses. The practice of consuming raw beef and conducting home slaughtering likely contributed to the high occurrence of bovine cysticercosis [15, 16].

Different studies were conducted in cattle slaughtered at Mekele and Bahir Dar municipal abattoirs, and other studies were also carried out in backyard slaughtered cattle at Wondogenet and Wolaita Soddo which showed that *Cysticercus bovis* and hydatid cysts were highly prevalent [17]. However, most studies done in Ethiopia focused mainly on the prevalence of foodborne zoonotic diseases and they did not indicate the knowledge and practice of livestock producers toward foodborne zoonotic diseases. Therefore, it is very important to evaluate the knowledge and practice of livestock producers toward foodborne zoonoses and their associated factors in and around Debre Tabor city. The objective of this study was to assess awareness of foodborne zoonotic disease and its associated factors in and around Debre Tabor city, Northwest Ethiopia.

## 2. Materials and Methods

### 2.1. Study Area

**Figure 1:**
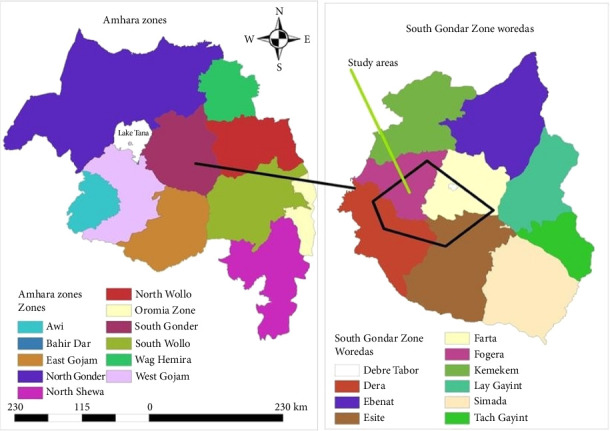
Map of Debre Tabor town, South Gondar Zone, Amhara Region, Ethiopia (created by QGIS Version 3.10.2 software).

This study was carried out in and around Debre Tabor city, Northwest Ethiopia (Figure 1). The study period was from January to June 2024. The area is located 100 km away from Bahir Dar and 664 km far from the capital city Addis Ababa. The city has six rural peasant associations and three subcities with an altitude from 1500 to 4231 MASL and 13°C–27°C temperature range. The annual rainfall ranges from 600 mm to 1200 mm. The total human population of the city is 201,787 (102,109 males and 99,678 females) with 39,882 households (31,485 males and 8397 females). Both governmental and private health and veterinary facilities exist in the city [8].

### 2.2. Study Design

A cross-sectional study was carried out to evaluate the awareness of foodborne zoonotic diseases and their associated factors in and around Debre Tabor city, Northwest Ethiopia.

### 2.3. Study Population

The study populations were those livestock-producer households living in and around Debre Tabor city.

#### 2.3.1. Inclusion Criteria

All livestock-producer households who were the heads of the family whose age was greater than 18 years were included in the study. In the absence of heads of the family, anyone from the house whose age was greater than 18 years was also included in the study.

#### 2.3.2. Exclusion Criteria

Family members whose age was greater than 18 but were seriously ill during the data collection period were not included.

### 2.4. Sample Size Determination and Sampling Procedures

The sample size was determined using the standard formula for estimating a single population proportion [18].

(1) $$\begin{array}{c} n=\frac{z_{\alpha /2}^{2}p\left(1-p\right)}{w^{2}}, \end{array}$$

where *n* = the sample size to be determined. *Z*_*α*/2_ = the standard normal distribution set at 1.96, which corresponds with the 95% confidence interval. P = population proportion that has good knowledge and practice. *q* = complements of *p* (i.e., 1 − *p*), i.e., population proportion that does not have good knowledge and practice. *w* = margin of error at 95% confidence level and since there was no information about *p* in the literature, we took *p* = 50% to get the maximum sample size. Assuming the required accuracy is 5%–10% for the nonresponse rate, we can substitute these values into the formula mentioned above.

(2) $$\begin{array}{c} \text{The sample size to be determined }\left(n_{1}\right)=\frac{{\left(1.96\right)}^{2}\times 0.5\times 0.5}{{\left(0.05\right)}^{2}}=384. \end{array}$$

Since the study population was less than 10,000 (i.e., *N* = 7497), we used finite population correction = *n*_1_/(1 + *n*_1_/*N*).

(3) $$\begin{array}{c} n_{2}=+=366. \end{array}$$

To account for a 10% nonresponse rate, we initially calculated the sample size as *n*_2_ = 366, which becomes *n*_3_ = 403 after adjustment. Doubling the sample size increases precision of the study. Therefore, we arrived at a final sample size of *n*_4_ = 806. Thus, the total number of participants was 806.

#### 2.4.1. Sampling Procedure

**Figure 2:**
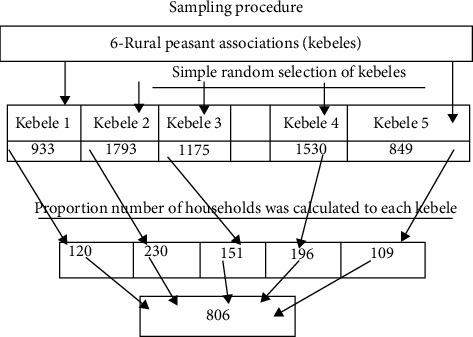
The number of households in each kebele was included as the study subject using a systematic sampling method.

There are six rural peasant associations (kebele) in the study area. Five peasant associations were selected randomly by using the simple random sampling method. From these randomly selected peasant associations, the livestock producers or participants in each peasant association were identified and selected randomly using a sampling frame available in the Debre Tabor city agricultural office. Then equal proportion of the sample size was distributed to the number of households in each peasant association. The numbers of households calculated in each peasant association were included in the study using a simple random sampling method (Figure 2).

#### 2.4.2. Data Collection Tools and Procedures

Closed-ended, structured questionnaires were developed. The questionnaires were initially prepared in English and then translated into Amharic by the principal investigator. They were retranslated back to English. The Amharic version of the questionnaire was used for data collection. Ten data collectors and two supervisors having educational levels of diploma and BSc were recruited from Debre Tabor city and trained for two days. The questionnaires were pretested in 30 households having similar characteristics to the study respondents, but they were not involved in the study. It was conducted one week prior to the actual data collection period. Data collectors were assigned to each peasant association by the investigator and then data were collected using self-administered multiple-choice and dichotomous questionnaires combined with interviews. Supervisors had carried out regular supervision, spot-checking and reviewing the completed questionnaires to maintain data quality. The principal investigator coordinated the overall activity.

### 2.5. Data Management and Analysis

Data manipulation was done using Epi Info Version 7 statistical software. The analysis was done by descriptive statistics and odds ratios. A backward stepwise binary logistic regression analysis was performed using SPSS software Version 17.0 to assess the significance of the explanatory variables. Logistic regression analysis was also used to see the effect of the different factors on the level of knowledge of foodborne zoonosis. The knowledge of foodborne zoonotic diseases was prepared in the form of binary variables (Yes = 1 and No = 0) and taken as the dependent variable while sociodemographic data and other independent variables are used. If the *p* value is < 0.05, it has been considered to represent a significant difference.

## 3. Results

### 3.1. Sociodemographic Characteristics

The overall response rate was 95.7% (771/806) in the study area. 553 (71.7%) males and 218 (28.3%) females participated. 33.1% of respondents were in the age group of 30–41 years. According to educational status, 227 (29.4%) noneducated and 332 (43.1%) elementary school respondents participated. Regarding their occupation, 548 (71.1%) respondents were farmers. 605 (78.5%) respondents were married, and based on their religion, most respondents were orthodox with a frequency of 671 (87%) from the total participants (Table 1).

### 3.2. Communities' Knowledge About Foodborne Zoonotic Diseases

679 (88.1%) participants responded that they had heard about foodborne zoonoses. 495 (72.9%) participants were males and 184 (27.1%) were females. The sources of information for respondents were training, magazines, books, leaflets/posters, friends/family, radio/TV, and school. 368 (33.5%) male and 153 (37.6%) female participants got information from friends or family, which was the most common source of information (Table 2).

### 3.3. Participants' Knowledge and Practice Related to Causes, Transmission, Prevention, and Control of Foodborne Zoonotic Diseases

192 (24.9%), 335 (43.5%), 313 (40.6%), and 266 (34.5%) respondents recognized TB, anthrax, taeniasis, and cysticercosis as foodborne zoonoses, respectively. 566 (73.4%), 620 (80.4%), and 514 (66.7%) participants understood that human beings might be infected with foodborne zoonotic diseases when they consumed foods from infected animals, unpasteurized milk, and raw meat, respectively. 705 (91.5%) and 627 (81.3%) respondents replied, respectively, that they could prevent and control foodborne zoonoses by taking care of animal health and by consulting veterinarians. 257 (33.3%) and 235 (30.5%) participants responded, respectively, that postmortem and antemortem inspections had been used for the prevention and control of foodborne zoonotic diseases (Table 3).

### 3.4. Level of Knowledge of the Community About Foodborne Zoonotic Diseases

**Figure 3:**
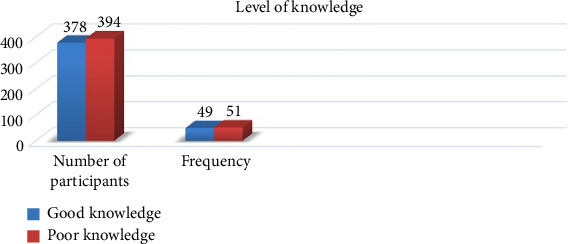
Level of knowledge of the community about foodborne zoonoses.

The level of knowledge of the community about the foodborne zoonotic disease in the study area was assessed by evaluating the response of each study participant. This was done by calculating the mean score of all respondents. Those study participants having results less than the mean score were categorized under poor knowledge (394 (51%)) and those having good knowledge greater than the mean score were grouped under good knowledge (378 (49%)) as shown in Figure 3.

### 3.5. Factors Associated With the Knowledge of Foodborne Zoonotic Diseases

The results of binary logistic regression analysis are described in Table 4. Educational status and access to information were statistically significant to good knowledge, while sex and age variables had no significant association with knowledge of foodborne zoonotic disease. High school (9–10 grades), preparatory school, and higher institutions had a significant association with knowledge of foodborne zoonotic diseases.

### 3.6. The Practices of the Community Toward Foodborne Zoonoses

676 (87.7%) respondents had explained that they had treated their animals in veterinary clinics. 376 (48.8%) respondents had been informed to avoid consuming the milk of lactating dairy cows, which were treated with antibiotics until the withdrawal period. 249 (32.3%) participants responded that they slaughtered sick animals everywhere in the field and consumed the raw meat. 421 (54.6%) participants purchased veterinary drugs without a prescription and 235 (30.5%) respondents sold sick animals' meat to the public. The practices of the community toward foodborne zoonotic diseases are described in Table 5.

### 3.7. Practice and Its Associated Factors Toward Foodborne Zoonotic Diseases

The overall results of the respondents having good and poor practice were 219 (41.4%) and 412 (58.6%), respectively (Table 6). Variables like sex, educational status, and practice status were significant, while other variables had no significant association with the practice of foodborne zoonotic diseases.

## 4. Discussion

The overall result of this study revealed that 394 (51%) and 219 (41.4%) respondents had poor knowledge and practice, respectively, about food bone zoonotic disease. The current finding was comparatively lower than the result of a study carried out in Addis Ababa and its surroundings [19]. This could be due to the variation in the sociodemographic character of the respondents. Most of the study respondents in the current study were farmers who were not accessible to information sources as we compared to the previous study repondants who were composed of health professionals [20]. The transmission of foodborne zoonotic diseases was more serious among the rural communities. This was because of their poor knowledge, traditional habit of consuming raw foods of animal origin, and having poor and unhygienic environment [21].

The prevalence of *T. saginata* infection in Ethiopia is high because of the common practice of consuming raw meat and the tendency to defecate in open pastures [22]. In Ethiopia, practices such as open pasture defecation, consuming raw beef or meat, and backyard slaughtering influenced the widespread occurrence of bovine cysticercosis [23]. This was due to having poor knowledge of foodborne zoonotic diseases [24]. Most of the respondents replied that human beings were infected with foodborne zoonotic diseases via foods from infected animals, unpasteurized milk, and raw meat consumption habits [25]. The frequency of respondents eating raw meat was high in the current study. This was due to the traditional habit of eating raw meat in the community. This finding agreed with a study conducted in Tanzania among animal health workers and livestock keepers having the habit of consuming raw meat which was still common practice especially in rural communities [26].

Even though the frequency of participants who had the habit of backyard slaughtering sick or healthy animals was low, the practice was very dangerous related to one health issue. The same was true for those respondents who sold sick animals' meat to the community. This could be due to the low level of awareness of the communities on the importance of using inspected meat [27, 28].

A high number of respondents reported that they sold milk either to their neighbors or to cafeterias. They bought veterinary drugs without any prescription from animal health professionals. Therefore, drug residue or drug withdrawal period in the milk was not under control. This led to public health effects and antimicrobial resistance [29, 30].

Educational status and access to information had a significant association (*p* < 0.05) with knowledge of foodborne zoonoses. Respondents having educational status of high school and preparatory had good knowledge about foodborne zoonotic diseases as compared to noneducated respondents [31].

Individuals having no access to information had poor knowledge about foodborne zoonotic diseases as compared to those individuals who had access to information [32]. Sex, educational status, and knowledge status of the respondents had a significant association (*p* < 0.05) with the practice of foodborne zoonoses [33]. Females had good practice toward foodborne zoonotic diseases as compared to males. This was because males usually participated in the backyard slaughtering of animals and consumed raw meat, which might increase their bad practices [34].

Respondents having adult educational status were less likely to have good practice toward foodborne zoonotic diseases as compared to noneducated participants [35]. This might be due to poor knowledge that could not bring a change in personal behavior. Respondents having educational status of high school, preparatory school, and higher institution had good practice toward foodborne zoonotic diseases as compared to noneducated participants [18, 36]. This finding agreed with the finding of a study in Addis Ababa and its surroundings [37].

## 5. Conclusion and Recommendations

The current study showed that most of the respondents had heard about foodborne zoonotic diseases from different sources of information; however, the knowledge status about foodborne zoonotic diseases was lower than the average level. The knowledge of respondents about the importance of postmortem and antemortem inspections was very limited, which are used as prevention and control measures for the transmission of the disease. Most of the respondents' perceptions about the disadvantages of eating raw meat and drinking milk without considering the drug residue withdrawal period were also very limited. Generally, the level of knowledge of respondents regarding foodborne zoonotic diseases was very poor. The level of good practice toward foodborne zoonotic diseases was lower than the level of knowledge; however, females had better practices than males toward foodborne zoonotic diseases. Educational status and access to information were very important predictor variables to increase the level of knowledge of the community about foodborne zoonotic diseases. Therefore, the following recommendations are forwarded:

i. An interdisciplinary collaborative approach between human health and animal health sectors could be practiced to promote the health of the community.
ii. Public education about foodborne zoonotic diseases shall be given regularly to the community.
iii. To lower the risk of food-borne zoonotic diseases, the government should set uniform regulations to regulate backyard type of slaughtering.

## Figures and Tables

**Table 1:** Sociodemographic characteristics of livestock producers in peasant association of Debre Tabor city, 2024 (*N* = 771).

Variables	Categories	Respondents	
		Frequency	Percent (%)
Sex	Male	553	71.7
	Female	218	28.3

Age (in years)	18–29	206	26.7
	30–41	255	33.1
	42–53	223	28.9
	> 53	87	11.3

Educational status	Noneducated	227	29.4
	Adult education	73	9.5
	Elementary school	332	43.0
	High school	103	13.4
	Preparatory and above	36	4.7

Marital status	Married	605	78.4
	Single	103	13.4
	Divorced	36	4.7
	Widowed	25	3.2
	Separated	2	0.3

Religion	Orthodox	671	87
	Muslim	79	10.2
	Protestant	19	2.8

Occupation	Farmer	548	71.1
	Merchant	41	5.3
	Government employee	14	1.8
	Housewife	84	10.9
	Other	50	6.5

**Table 2:** Respondents' information accessibility about foodborne zoonoses and their sources at Debre Tabor city (*N* = 771).

Variables	Sex of respondents			
	Categories	Male *N* (%)	Female *N* (%)	Total *N* (%)
Access to information to foodborne zoonoses	Yes	495 (64.2)	184 (23.9)	679 (88.1)
	No	58 (7.5)	34 (4.4)	92 (11.9)

Sources of information	Training	219 (19.9)	72 (17.7)	291 (19.3)
	Magazine	68 (6.2)	19 (4.7)	87 (5.8)
	Books	95 (8.7)	26 (6.4)	121 (8)
	Leaflets/poster	18 (1.6)	8 (2)	26 (1.7)
	Friends/family	368 (33.5)	153 (37.6)	521 (34.6)
	Radio/TV	185 (16.8)	75 (18.4)	260 (17.3)
	School	145 (13.2)	54 (13.3)	199 (13.2)

**Table 3:** Participant's knowledge and practice related to causes, transmission, prevention, and control of foodborne zoonotic diseases.

Variables	Sex of respondents			
	Responses	Male *N* (%)	Female *N* (%)	Total *N* (%)
*Foodborne zoonoses*				
• TB	Yes	120 (15.6)	72 (9.3)	192 (24.9)
	No	433 (56.2)	146 (18.9)	579 (75.1)

• Anthrax	Yes	269 (34.9)	66 (8.6)	335 (43.5)
	No	284 (36.8)	152 (19.7)	336 (56.5)

• Cysticercosis	Yes	245 (37.8)	68 (8.8)	313 (40.6)
	No	308	150 (19.5)	458 (59.4)

• Taeniasis	Yes	179 (39.9)	87 (11.3)	266 (34.5)
	No	374 (48.5)	131 (17)	505 (65.5)

*Sources of infection*				
• Food from an infected animal	Yes	417 (54.1)	149 (19.3)	566 (73.4)
	No	136 (17.6)	69 (8.9)	205 (26.6)

• From unpasteurized milk	Yes	441 (57.2)	179 (23.2)	620 (80.4)
	No	112 (14.5)	39 (5.1)	151 (19.6)

• From raw meat	Yes	356 (46.2)	158 (20.5)	514 (66.7)
	No	197 (25.6)	60 (7.8)	257 (33.3)

*Experience of infection*				
• Ever been sick when you consumed foods of animal origin	Yes	315 (40.9)	126 (16.3)	441 (57.2)
	No	238 (30.9)	92 (11.9)	330 (42.8)

*Prevention and control mechanisms*				
• Keeping animal health	Yes	507 (65.8)	198 (25.7)	705 (91.5)
	No	46 (6)	20 (2.6)	66 (8.6)

• By consulting veterinarians	Yes	451 (58.5)	176 (22.8)	627 (81.3)
	No	102 (13.2)	42 (5.5)	144 (18.7)

• By postmortem inspection	Yes	186 (24.1)	71 (9.2)	257 (33.3)
	No	367 (47.6)	147 (19.1)	514 (66.7%)

• By antemortem inspection	Yes	181 (23.5)	54 (7)	235 (30.5)
	No	372 (48.2)	164 (21.3)	536 (69.5)

**Table 4:** Sociodemographic predictors of knowledge toward foodborne zoonoses and its logistic regression analysis in Debre Tabor city.

Variables	Frequency		Odds ratios	
	GK	PK	COR (95% CI)	AOR (95% CI)
*Sex*				
• Male	267	287	0.032 (0.02, 0.60)	0.02 (0.01, 0.03)
• Female	111	107	1.46 (1.03, 1.95)	1.2 (1.1, 1.7)

*Age (in years)*				
• 18–29	116	90	1.0 (0.9, 1.5)	0.8 (0.9, 1.3)
• 30–41	137	118	0.85 (0.59, 1.24)	0.6 (0.4, 1)
• 42–53	102	121	0.64 (0.44, 0.94)	0.4 (0.5, 0.8)
• > 53	22	65	0.37 (0.22, 0.62)	0.2 (0.3,0.5)

*Educational status*				
• Noneducated⁣^∗^	88	139	1.0	
• Adult education	23	50	1.09 (0.64, 1.85)	1.06 (0.61, 1.85)
• Elementary school (1–8)	165	167	1.64 (1.16, 2.30)	1.34 (0.93, 1.93)
• High school (9–10)	58	95	2.56 (1.58, 4.15)	1.99 (1.20, 3.34)⁣^∗∗^
• Preparatory & above	23	13	14.50 (4.32, 48.59)	10.16 (2.99, 34.52)⁣^∗∗^

*Access to information*				
• Yes⁣^∗^	350	329	1.0	
• No	16	76	0.15 (0.08, 0.260)	0.18 (0.10, 0.31)⁣^∗∗^

Abbreviations: AOR = adjusted odds ratio, COR = crude odds ratio, GK = good knowledge, PK = poor knowledge.

⁣^∗^Reference category.

⁣^∗∗^Significant association (*p* < 0.05).

**Table 5:** Participants' good and bad practices toward foodborne zoonotic diseases.

Variables	Responses	
	Yes	No
• Have you ever had your animal treated in veterinary clinics?	676 (87.7%)	95 (12.3%)
• Have you ever been told by animal health workers about the milk of newly treated lactating cows with antibiotics?	376 (48.8%)	395 (51.2%)
• Have you encountered any public conferences discussing foodborne zoonotic diseases?	278 (36.1%)	493 (63.9%)
• Have you ever participated in any training about foodborne zoonotic diseases?	218 (28.3%)	553 (71.7%)
• Slaughtering sick animals in the field and consuming the meat with others in the field	49 (32.3%)	522 (67.7%)
• Purchasing veterinary drugs without a prescription	421 (54.6%)	350 (45.4%)
• Selling of sick animals' meat to the public	235 (30.5%)	536 (69.5%)
• Sell milk to cafeterias	309 (40.1%)	462 9.9%)

**Table 6:** Predictor variables associated with the practice of foodborne zoonoses, Debre Tabor city, 2014.

Variables	Frequency		Odds ratios	
	GP	PG	COR (95% CI)	AOR (95% CI)
*Sex*				
• Male⁣^∗^	193	360		
• Female	126	92	0.39 (0.28, 0.54)	2.17 (1.53, 3.07)⁣^∗∗^

*Educational status*				
• Noneducated⁣^∗^	82	145		
• Adult education	17	56	0.54 (0.29, 0.99)	0.52 (0.28, 0.87)⁣^∗∗^
• Elementary school (1–8)	123	209	1.04 (0.73, 1.48)	0.85 (0.59, 1.24)
• High school (9–10)	65	38	3.03 (1.87, 4.90)	2.21 (1.33, 3.69)⁣^∗∗^
• Preparatory and above	32	4	14.15 (4.83, 41.41)	7.32 (2.45, 21.85)⁣^∗∗^

*Practice status*				
• Poor practice⁣^∗^	92	263	5 (4.5, 8)	4 (3, 6.5)
• Good practice	227	189	3.43 (2.53, 4.66)	2.98 (2.15, 4.11)⁣^∗∗^

Abbreviations: AOR = adjusted odds ratio, COR = crude odds ratio, GP = good practice, PP = poor practice.

⁣^∗^Reference category.

⁣^∗∗^Significant association (*p* < 0.05).

## Data Availability

The data that support the findings of this study are available from the corresponding author upon reasonable request.

## Nomenclature

AOR: Adjusted odds ratio
BTB: Bovine tuberculosis
COR: Crude odds ratio
CSA: Central statically agency
FAO: Food and Agriculture Organization
OIE: Organization for Animal Health
WHO: World Health Organization
